# Social differentiation and embodied dispositions: a qualitative study of maternal care-seeking behaviour for near-miss morbidity in Bolivia

**DOI:** 10.1186/1742-4755-6-13

**Published:** 2009-07-29

**Authors:** Mattias Rööst, Cecilia Jonsson, Jerker Liljestrand, Birgitta Essén

**Affiliations:** 1 International Maternal and Child Health (IMCH), Department of Women's and Children's Health, Uppsala University, Sweden; 2 School of Health Sciences and Social Work, Växjö University, Sweden; 3 Faculty of Medicine, Lund University, Sweden

## Abstract

**Background:**

Use of maternal health care in low-income countries has been associated with several socioeconomic and demographic factors, although contextual analyses of the latter have been few. A previous study showed that 75% of women with severe obstetric morbidity (near-miss) identified at hospitals in La Paz, Bolivia were in critical conditions upon arrival, underscoring the significance of pre-hospital barriers also in this setting with free and accessible maternal health care. The present study explores how health care-seeking behaviour for near-miss morbidity is conditioned in La Paz, Bolivia.

**Methods:**

Thematic interviews with 30 women with a near-miss event upon arrival at hospital. Near-miss was defined based on clinical and management criteria. Modified analytic induction was applied in the analysis that was further influenced by theoretical views that care-seeking behaviour is formed by predisposing characteristics, enabling factors, and perceived need, as well as by socially shaped habitual behaviours.

**Results:**

The self-perception of being fundamentally separated from "others", meaning those who utilise health care, was typical for women who customarily delivered at home and who delayed seeking medical assistance for obstetric emergencies. Other explanations given by these women were distrust of authority, mistreatment by staff, such as not being kept informed about their condition or the course of their treatment, all of which reinforced their dissociation from the health-care system.

**Conclusion:**

The findings illustrate health care-seeking behaviour as a practise that is substantially conditioned by social differentiation. Social marginalization and the role health institutions play in shaping care-seeking behaviour have been de-emphasised by focusing solely on endogenous cultural factors in Bolivia.

## Background

A 75% reduction in maternal mortality worldwide by 2015 is one of the millennium development goals and a priority in global public health. Skilled birth attendance is regarded as a key factor in reducing maternal mortality, primarily through prevention of infections and haemorrhage, and secondarily by recognising and acting upon possible complications in a timely manner [1]. In the event of the latter, the availability of emergency obstetric care is fundamental, although availability alone is insufficient to ensure real access.

In maternal health research, accessibility (as influenced by infrastructure, distance to maternal health facilities, and the cost of maternal care) has been stressed as the main factor determining the use of antenatal care, skilled birth attendance, and emergency obstetric treatment [2-4]. Some studies have shown that increased health service accessibility significantly reduces the socioeconomic differentials in maternal care-seeking [5,6]; other studies, however, have concluded that accessibility has little effect [7]. Hence, utilisation is not simply ensured by availability and accessibility, but has increasingly been viewed as a behavioural phenomenon, with research directed at characterising the motivations and actions of pregnant women [6,8].

Theoretical models of health care-seeking behaviour have focused predominantly on describing a series of stages and related actions from the initial perception of symptoms to the utilisation of medical assistance, and on determinants that influence such actions [9,10]. The utilisation of maternal health care in low and middle-income countries has been statistically associated with several socioeconomic and demographic factors, such as maternal education, female autonomy, marital status, parity, and health insurance [7,8,11-15]. However, maternal employment and ethnic background have shown contradictory patterns [7,15-17]. Seeking professional health care for complications has also been associated with maternal age, urban residence, and low parity [18]. Although these determinants might function differently in varying settings, there have been few theoretical discussions about how such utilisation is shaped within a local context.

The Bolivian government's main policy in addressing high levels of maternal mortality has been to increase accessibility by reducing financial barriers. Maternal health care has been provided without cost since 1996 through a government-subsidized program now known as SUMI (Seguro Universal Materno-Infantil, Universal Maternal and Child Insurance). SUMI provides health care for pregnant women, including antenatal checkups, delivery, caesarean section, intensive care, postpartum follow-ups, and family planning for six months after delivery [19]. Since the implementation of the SUMI program there are indications of a reduction in maternal mortality in Bolivia. According to national figures based on household surveys, the maternal mortality ratio (MMR) was estimated at 390/100,000 live births in the year 1994 and at 229/100,000 in 2003 [20,21]. After modelling for methodological uncertainties the latter figure was, in the World Health Organisation's estimates, upgraded to 290/100,000 [22]. However, the wide range of uncertainty (160–430/100,000) makes MMR trend assessments difficult. Uptake of maternal health care has increased with a rise in skilled birth attendance from 47% in 1994 to 61% in 2003, and attendance to antenatal care improved from 53% to 79% [20,21]. However, this increased utilization of maternal health care has stagnated in this decade, and the vast majority of poor and low educated Bolivian women continue to give birth at home without skilled birth attendance [21]. Furthermore, a study of severe obstetric morbidity (near-miss) at hospital level in urban Bolivia, conducted by the authors of this paper, showed that late hospital arrival in cases of obstetric complications is common, underscoring the significance of pre-hospital barriers, even where free maternal health care is readily obtainable within reasonable distances [23]. Thus, one is led to consider determinants other than cost and geographical proximity as factors in gaining access to maternal health care.

By means of thematic interviews with women who have experienced severe obstetric complications (near-miss), this study explores the way health care-seeking behaviour is conditioned in an urban Bolivian setting that offers free and easily accessible maternal health care.

## Methods

### Study setting

This study employed a qualitative approach and was part of a larger project that identified all near-miss cases and maternal deaths at four maternity hospitals in the La Paz district of Bolivia over a period of six months (from September 2006 to February 2007). Near-miss was defined as a life threatening pregnancy-related complication that was resolved by chance or medical intervention. Clinical and management-based criteria were used to identify near-miss cases within five major diagnostic groups; *severe haemorrhage *resulting in shock, hysterectomy or multiple blood transfusions; *severe hypertensive disorders *including eclampsia or severe preeclampsia, the latter defined as high blood pressure (>140/90 mmHg, or an increase >30/15 mmHg from baseline) with neurological symptoms and either proteinuria of >1 g/24 h or laboratory findings consistent with HELLP (haemolysis, elevated liver enzymes, and low platelets); *sepsis *(hypo/hyperthermia with clinical signs of shock); *obstructed labour *including uterus rupture or impending rupture (clinical signs of Band's ring or surgical findings); *severe anaemia *with haemoglobin corresponding to <6 g/dl at sea level (<9 g/dl in La Paz, and <9.5 g/dl in El Alto) with tachycardia or dyspnoea [23]. Two of the hospitals in the study are designated as first referral level hospitals in El Alto; two are second referral level hospitals in La Paz, the administrative capital of Bolivia. El Alto, until recently described as a suburb of La Paz, had a population of 11,000 inhabitants in the 1950s. In the last thirty years it has rapidly expanded as a result of rural-urban migration and is today an autonomous urban area with an almost exclusively indigenous population and great poverty [24]. Today La Paz and El Alto each has a population of approximately 800,000 inhabitants. Both of these urban settings are characterised by the relatively high availability of maternal health-care facilities, short distances to hospitals, abundant public transportation, and a network of urban ambulances. Nevertheless, institutional deliveries in the La Paz district are generally less common than in Bolivia as a whole. Only 41% of all women in the district give birth at a health-care facility, whereas 59% deliver at home, usually attended by family members [21].

### Population and data collection

Semi-structured interviews consisting of open-ended questions were conducted with 30 women who had experienced a near-miss event. Informants were strategically selected based on three premises that we found important for the research objective. First, informants were chosen among women with a near-miss event upon arrival at the hospitals, as these women have generally experienced pre-hospital barriers [25]. Second, the women interviewed had survived one of the most common causes for near-miss in the area (severe haemorrhage or severe hypertensive disorders) [23]. Third, we sought an inclusion of women with different socio-demographic backgrounds. Each interview lasted from 45 to 60 minutes and was conducted in Spanish on the hospital premises by the first author after the women had recuperated from their complications. The interview setting aimed at establishing confidentiality and trust. In accordance with the preferences of most women, no interviews were recorded. Notes were taken and carefully revised immediately after the interviews, at which time they were also augmented by descriptions, impressions, and quotations. The quotations presented in this paper reflect the essence of an informant's description, but may not always be verbatum. Informants were encouraged to speak freely about what they had experienced, beginning with the onset of their complications up to the time of the interview. Attention centred on the following themes: the informant's health-care experiences during prior pregnancies, her antenatal care, her family's delivery traditions, her preferences regarding where to deliver, her knowledge of the SUMI program, and any other considerations or actions taken at the time complications set in.

### Data analysis

The narratives of the 30 informants and their perceptions of maternal health care were coded and analysed by two researchers, one with a medical and the other with a sociological background. The analysis was influenced by the procedures of analytic induction that seeks to elaborate and refine explanations of a phenomenon through a systematic search for contradictory evidence [26]. Consequently, analysis was a continuous process throughout data collection. This method was originally developed to establish universally applicable patterns of causality. In the present study it was employed to identify and explain patterns of behaviours within our setting, an approach sometimes referred to as modified analytic induction [27]. Theoretical saturation (i.e. no further substantial information discerned) was experienced after about 20 interviews. As we found no evidence that contradicted our evolving hypothesis when the interview series was continued, we concluded data collection after 30 informants. Our first analytic stage sought out commonalities in the experiences and perceptions of women in the cohort in order to identify and classify general patterns. The following focused on information that deviated from the general patterns, both in order to decrease the risk of overinterpreting the data and as a means of testing the explanatory validity of our findings.

### Theoretical framework

Determinants for use of maternal health care can be conceptualised by applying a behavioural model proposed by Andersen that seeks to account for and predict the use of health services by individuals [28]. According to the model, such utilisation is dependent on the interaction between individual traits, population characteristics, and the surrounding environment. Andersen proposes that the relevant factors can be grouped into three main categories: an individual's *predisposition *to use medical services; *enabling or impeding circumstances *(such as infrastructure); and the *need *for health care. Predisposing characteristics are related to demographic elements and social structure, including age, gender, residence, occupation, education, ethnicity, and attitudes toward health. Enabling elements consist of community factors that affect the availability and accessibility of health care, and personal factors such as knowing how to take advantage of what is offered. Finally, characteristics associated with need include types of illness, perceived health status, and expected outcome of treatment. In the context of the present study, "need" refers to an informant's perceived need of maternal health care.

Most theoretical models view health care-seeking behaviour as a result of rational individual choice. As such, they have been criticised for giving inadequate attention to the social context within which actions are taken by individuals [9]. In attempting to conceptualise patterns of predisposing characteristics, our analytical framework has been influenced by the social theory of Bourdieu in which the relationship between individuals and structure is described. Central to this perspective is the concept of habitus as the embodied dispositions to which people resort as a framework for their perceptions and actions. The theory assumes that social structures provide access to different conditions (i.e., social, cultural, and economic capital) and that habitus is a result of constant exposure to these conditions from a certain relative position within a particular context [29]. Bourdieu's view has been used to theorise inequalities in health and illness [30]. In some instances it has been applied to behavioural aspects of childbirth and institutional change within obstetric care [31,32]. In the analysis below, we avail ourselves of this theory to examine how patterns of maternal health-care utilisation and inequalities in accessibility are products of accumulated dispositions.

### Ethical considerations

This study was approved in 2006 by the Bolivian National Committee for Bioethics: Commission for Ethics in Investigations (Comite Nacional de Bioetica: Comisión de Ética de la Investigación). All interviews were conducted after the study's aim was explained to the participants and their informed consent was obtained. It was emphasised that participation was voluntary, that the interviewer was not a member of the government or the hospital staff, and that the identity of all informants would remain confidential.

## Results

### Characteristics of informants

The 30 informants in the study constituted a heterogeneous group regarding age, marital status, levels of education, places of residence, and parity, as was also the case of all women surveyed with near-miss (Table 1). The informants ranged from ages 18 to 42. The majority (n = 24) were married or lived with a partner. Approximately half of the women were pregnant for the first time (n = 12) or had previously been pregnant from 1 to 4 times (n = 14). Six women lived in rural areas, while the others lived in La Paz (n = 8) or in El Alto (n = 16). The majority (n = 17) had attended fewer than the recommended four antenatal care appointments. All informants had arrived at hospital in critical condition, fulfilling the criteria for near-miss, and had survived a severe haemorrhagic complication, severe preeclampsia, or eclampsia.

**Table 1:** Background characteristics of selected informants compared to all women with severe maternal morbidity (near-miss) at four hospitals in the La Paz region of Bolivia, September 2006 to February 2007 (valid percentage).

Characteristics	Informants n = 30	Near-miss total n = 401
Age (years)		
< 20	3 (10)	52 (13)
20–35	22 (73)	278 (70)
> 35	5 (17)	69 (17)
Missing	0	2
Mean (SD)	29 (6.6)	28 (7.1)
		
Marital status		
Single	6 (20)	45 (12)
Co-habiting	24 (80)	335 (88)
Missing	0	21
		
Education		
No formal education	0	13 (3)
Primary (8 yrs)	15 (50)	136 (38)
Secondary (12 yrs)	12 (40)	161 (45)
Higher (> 12 yrs)	3 (10)	50 (14)
Missing	0	41
		
Residency		
La Paz	8 (27)	137 (34)
El Alto	16 (53)	193 (48)
Rural	6 (20)	70 (18)
Missing	0	1
		
Parity		
0	12 (40)	118 (30)
1–4	14 (47)	224 (56)
> 4	4 (13)	57 (14)
Missing	0	2
Median (Range)	2 (0–7)	2 (0–12)
		
Antenatal control		
0	9 (30)	100 (27)
1–3	8 (27)	95 (25)
> 3	13 (43)	179 (48)
Missing	0	27
Median (Range)	2.5 (0–9)	3 (0–16)

### Strategies shaped by family traditions and composed experiences

The informants in this study can be divided into two distinct groups, each exhibiting different attitudes toward maternal health care and each reflecting their social background. How they planned to deliver and the actions they took when a severe complication arose clearly delineated the two groups. Women in the first category planned to deliver at hospital, generally lived in La Paz, and were conversant with the health-care system. They all had experience of maternal health care through antenatal checkups or in earlier pregnancies. The second category, women who planned to deliver at home, lived in rural areas or in the recently urbanized El Alto. They had comparatively low socioeconomic status, as judged by their resources and educational level. Almost without exception, they had no personal or family experience of antenatal visits or obstetric care. The plan of delivery of women in both categories coincided with their traditions and family experiences: few in either group questioned their assumptions with regard to childbirth. Regardless of their plan, almost no one said they had weighed other possibilities. Those women for whom delivery at home was customary told of how they intended to give birth matter of factly. This is illustrated in the following two statements by women who had never given a thought to the possibility of delivering in a hospital:

We [poor people] give birth at home. That's just how we do it (Informant 5, El Alto). We're walking around the patio and then [a minute later] we give birth alone. That's simply the way it is (Informant 10, Rural).

These women consistently referred to themselves as belonging to a *group *of women who follow a tradition of giving birth at home. Consequently, descriptions of a choice being made between home or hospital deliveries, with positive expectations weighed against negative ones, were not encountered in their narratives. Rather, they emulated the behaviour of the group they identified most closely with.

Family traditions were also important for those women who planned to deliver in a hospital. Women in this group often described a more reflective decision making process informed by their family's previous experiences, which also guided them in choosing which hospital to deliver in. For them, birthing at home was out of the question:

My family told me that it is better to give birth in a hospital than at home (Informant 11, La Paz). My sister gave birth here [at this hospital] and said this was a good hospital and that the staff was nice(Informant 30, La Paz).

The only two deviations from family patterns were one woman who had been advised to deliver at hospital due to placenta previa, and one who had had such a negative personal experience with a previous home delivery that she decided on her own that it was safer to give birth in a hospital.

A woman's initial preference with regard to maternal health care also influenced her care-seeking behaviour when complications arose. Those women oriented towards delivering at home generally sought medical intervention later than women who preferred hospital deliveries. In the case of those favouring home birthing, we consistently noted a pattern of delaying the decision to seek emergency care until they themselves, or a close relative, perceived their symptoms as directly life threatening. A woman with placenta previa who arrived in shock explained what had happened before she came to the hospital:

I was doing the laundry when I suddenly started to bleed. First a little, then more and more, until finally I was bleeding all the time. I didn't know what to do and went to bed, hoping it would just stop by itself(Informant 21, El Alto).

When she became unconscious, the woman's husband and sister brought her to the nearest health-care centre, from which she was immediately transferred to a hospital. The majority of women in her category took the final decision to seek emergency care after discussions with their family, or ended up being transported to the hospital in an unconscious state by relatives. Family members played an especially important role in advising women with no previous experience of maternal care how to act on their symptoms when complications arose.

Women experiencing a sudden onset of severe bleeding or eclamptic seizures who were not alone at the time were rushed to the hospital by family members. However, the majority of the women with severe preeclampsia did not consider what was occurring to them severe enough to seek immediate medical attention. Rather, they told of waiting for the symptoms to pass. Some said they initially thought that what they were experiencing was normal in pregnancy, while others felt uncertain about the significance of the signs.

I had a headache, strange sounds in my ears, and I saw lights in front of my eyes. But I didn't think that it could be dangerous (Informant 8, El Alto).

Surprisingly, among those of our informants who had had regular antenatal checkups, none reported that danger signs in pregnancy had ever been discussed with them during such visits.

### The perception of not belonging

Women oriented towards home deliveries appeared totally disconnected from the health-care system. They stated that hospitals were "not for them" and admitted having only a vague notion of what a hospital delivery involves:

We know nothing about hospitals, so we give birth at home (Informant 13, El Alto). We don't know what goes on in hospitals, so I just thought that I should give birth at home (Informant 20, El Alto).

Women favouring home birth saw themselves as outsiders, a view they said their group shared. They referred to each other in collective terms, saying things such as "we do not belong there". When asked to explain why they had not considered giving birth within the health-care system, these women cited hearsay evidence that centred on two aspects of delivering in a hospital. First, they described hospitals as dangerous places where one ran the increased risk of serious complications. For example, they were afraid of negative consequences arising from caesarean sections, perceiving them as risky and possibly life-threatening. They also worried that a caesarean section would be performed on them for reasons they did not understand, and feared the procedure would interfere with their ability to work in the future. Second, some informants had heard that women are not well treated in hospitals and are not told anything about their condition.

They [family and friends] say that it is dangerous to be operated on, that you could die because the scar often bursts, and things like that (Informant 26, El Alto.) I didn't want to deliver in a hospital. It would be a bad experience and you can get diseases by delivering in a hospital (Informant 9, El Alto). I wanted to deliver at home, where your family takes care of you right away. It is not like that in the hospital, where there are delays [when you call for someone] and sometimes you are treated badly. They [family and friends] also told me that there is a higher risk of your baby dying in a hospital. You are not taken care of well, and sometimes women die in hospitals (Informant 21, El Alto).

None of the informants cited cultural practices (such as the tradition of burying the placenta or using herbal medicines) as reasons for preferring home deliveries.

With regard to personal enabling factors, we found that approximately half of our informants were unfamiliar with the SUMI program that provides maternal health care free of charge. These were primarily women who intended to give birth at home. The worries some informants expressed about the hospital bill they would receive after being discharged were epitomised by one woman as follows:

I haven't paid anything so far, but I worry about what they will charge me for my stay when I leave the hospital (Informant 4, Rural).

One woman, despite being told about SUMI by the interviewer, remained sceptical and asked:

But will it cover [my expenses] even if I have had surgery? [caesarean section] (Informant 10, Rural).

Although cost did not figure as a major obstacle in seeking health care at the time of complications, it did in non-emergency situations. Perceived cost was described by some women as directly influencing their decision not to have regular antenatal checkups or arrange for delivery within the health-care system. Our informants were clearly divided in their awareness of the SUMI program. Those who lacked this knowledge lived in rural areas or in El Alto and had planned to deliver at home. Conversely, women familiar with SUMI had generally heard about it through contacts with the health-care system during previous pregnancies or at the time of antenatal checkups.

### Mistreatment and distrust

There were similarities in informants' impressions about their hospital stay. Mistreatment by staff was a recurring theme.

The staff is harsh. They don't listen to you and they don't explain anything that they're doing. You just lie alone in your bed feeling scared and lonely. You don't know why you can't go home or what they are doing to you (Informant 8, El Alto).

However, positive attitudes toward hospital delivery appeared not to be shaken as a result of such negative experiences. Women who had initially planned to deliver at hospital stated that in the event of future pregnancies they would continue to avail themselves of institutional health care, although they would choose another hospital. Similarly, women influenced by tradition to deliver at home were reinforced in their preference, saying that if they were to have another child, they would definitely want to give birth at home. Informants commonly complained about the lack of information the hospital staff gave them regarding their complications, and how little they were told of the treatment they were being given.

I don't know what happened. They only told me that I bled and that my baby was dead (Informant 25, Rural). I'm very worried because I don't know what happened to my baby. They took him to another room and then I don't know what happened (Informant 3, Rural). They say I have to stay in the hospital, but I don't understand why because I feel much better now and want to go home (Informant 12, El Alto).

A dread of caesarean sections and hospitalisation resulted in three women initially refusing surgical interventions, delaying their recovery. Fear of dying and failing to understand the rationale for an invasive procedure were the main reasons for such refusal. Some women worried about the consequences of the caesarean section they had already undergone. This was exemplified by one informant who lamented that her fears of being sterilized if she went to a hospital came true because she had now had a caesarean section. She said she did not dare ask the hospital staff for further information.

## Discussion

Our analysis identifies two categories of women, each having separate preferences for maternal health care: one is conversant with the health-care system, and the other is dissociated from it. For women in both categories, care-seeking behaviour was related to traditions and composite experiences within their families. We found that reflective decision making was rare. Among women who felt excluded from the health-care system, distrust and fear of surgical intervention were common. These women generally lacked knowledge of the SUMI program. None of our informants cited specific cultural practices, such as delivering with a traditional birth attendant or using traditional medicine, as a reason for preferring to deliver at home, nor were such practices mentioned to account for their delay in going to the hospital when complications arose.

### Strengths and limitations

Mortality reviews and verbal autopsies have been the most common approaches in investigating barriers to maternal health care in developing countries [33]. Focusing on near-miss events provides the methodological advantage of having firsthand information from surviving women in trying to understand maternal health care-seeking behaviour. In the present study, informants were deliberately chosen from women with a near-miss event upon arrival at hospital, as they represent a group that generally have undergone pre-hospital delays in obtaining emergency obstetric care [25].

One limitation of facility-based studies is that only women who reach hospitals are included, as our results reflect. In our view, the low impact of cost on health care-seeking behaviour at the time of the complication could be a reflection of sampling, since women experiencing significant financial barriers might not be represented in this study. Consequently, to reach a complete picture of pre-hospital barriers our findings need to be complemented by community-based research.

### Social differentiation and embodied dispositions

Women with a low socio-economic status are less prone to use formal maternal health care services [34]. Although, such services are provided without cost in Bolivia, inequalities in utilization are the largest in the world. Only 27% of women in the lowest income quintile use skilled birth attendance as compared to 98% in the highest income quintile. Similar figures are seen if one compares women with the lowest and highest educational levels (30% vs. 98%) [21]. Several studies have described how barriers related to single socio-demographic factors can impede accessibility to maternal health care, such as distance for women living in rural areas or indirect costs of care and transportation for poor women [35]. Such mechanisms are probably most important also in Bolivia. The present study adds to this understanding of socio-demographic inequalities in utilization of maternal health care by showing that care-seeking behaviour corresponds to how women perceive their relative position within their social world. Within our setting variations in maternal care-seeking behaviour were closely related to social differentiation in the self-perception of women who held a shared understanding of being fundamentally separated from "others", e.g., belonging to a social category that did utilise health care. Thus, it illustrates that care-seeking is a socially structured practice that operates on the level of internalized logic, rather than on reflective decision making by the women [29]. This Bourdieusian interpretation challenges models that describe utilization of health care as mainly a result of rational choice. Rather, it highlights care-seeking as being part of schemes of perceptions and actions that correspond to embodied dispositions that in turn are shaped by combined socio-demographic disadvantages.

Interestingly, women living in El Alto and in rural areas described similar perceptions of being dissociated from the health-care system. Although the mechanisms behind this needs to be further investigated in future studies, our interpretation implies that the context of informality and low affiliation with public services that is part of these women's everyday life also is a strong factor in whether they use maternal health care [24].

The present study further supports the importance the factor of perceived need plays in determining care-seeking behaviour, as proposed by Andersen [28]. This is exemplified by the women who only sought health care when their situation changed so dramatically that it was seen as potentially life threatening. This illustrates that practice corresponding to embodied dispositions are not fully predetermined, but may be subject to change. Increased use of skilled birth attendance and early recourse to medical assistance for obstetric complications is vital to reduce the high levels of maternal mortality and morbidity in Bolivia. Our results underscore that bringing about such a change will require that initiatives to decrease specific barriers, such as cost and distance, are combined with strategies to reduce social marginalization.

### Improving personal enabling resources

The low awareness of the SUMI program among women favouring birthing at home illustrates the unequal distribution of personal enabling resources in Bolivia. Early evaluations of the government subsidized program showed that a large part of the population, especially in rural areas, were unfamiliar with their entitlement to free health care [36,37]. Continuing governmental efforts to disseminate information about SUMI have included media campaigns in radio, television and newspapers. For those already in contact with the health care system the program has been promoted by staff and posters in most health-care facilities. Further, mobile health teams have been assigned to increase coverage of services provided within the SUMI program in remote areas. However, lack of knowledge about the benefits of the program has also recently been described in another qualitative study [38]. Although there are no current figures on how widespread knowledge about the program is, our results indicate ongoing difficulties to reach women who are not already using maternal health care. Future empirical studies are required to determine to which extent lack of such knowledge directly influences maternal health in Bolivia.

### Making health care trustworthy

Lack of confidence in the health-care system, hearsay about negative outcomes, and reports of poor treatment by hospital staff were commonly encountered in this study–phenomena that have also been described in other settings [38,39]. We argue that such perceptions conceptualise and inform a group's common behaviour, gradually becoming incorporated in an embodied disposition that, on the one hand, is shaped by social differentiation and, on the other, further drives the process of reproducing it. Distrust in the health-care system was increased by perceptions of being mistreated and deprived of essential information, reinforcing preferences for home delivery. These factors strongly influence utilisation of antenatal and delivery care, as well as emergency obstetric care [40,41]. Fear of caesarean sections, often rooted in distrust of medical practitioners, has been described as a major reason for women avoiding maternal care, with potentially devastating consequences by delaying essential surgery [42-44]. The role health institutions play in shaping care-seeking behaviour needs further attention and it should be acknowledged that it is partly their responsibility to increase women's confidence in the services provided in order to counteract inequalities in utilisation.

### Cultural barriers or socially structured disadvantages?

Low use of reproductive health services in Latin America–particularly Bolivia–is often ascribed to the fact that the biomedical health model discounts cultural influences among indigenous populations [38,45]. Almost all women in rural areas and in El Alto have an indigenous background and are less likely to use skilled birth attendance, compared to the national average [21]. However, the present study found no evidence in the narratives of informants that preferences for cultural practices played a role in preventing or delaying women from seeking maternal health care. As the indigenous population in Bolivia is especially subject to social exclusion [46], it is open to question whether the low utilisation of maternal health care is as closely related to cultural preferences as has been previously thought, or if it is mostly a result of socially structured disadvantages related to ethnic background. This distinction has major policy implications, since reducing the problem to endogenous cultural factors in a simplistic way tends to obscure other contextual determinants and blame the failure to access health care on women themselves [47].

## Conclusion

The present study adds to the understanding of socio-demographic inequalities in utilization of maternal health care by illustrating that a perception of being dissociated from such services, lack of personal enabling resources and distrust are integrated in shaping maternal care-seeking behaviour. This indicates a need to complement initiatives that decrease specific barriers related to cost and distance with strategies that reduce social marginalization. We further suggest that a one-sided focus on endogenous cultural factors has de-emphasised the importance of social marginalisation and the role health care institutions play in the discussion of why the benefits of the SUMI program have been unable to reach a large part of the population in Bolivia.

## Competing interests

The authors declare that they have no competing interests.

## Authors' contributions

MR conceived the idea for the study, conducted the interviews, and together with CJ and BE is responsible for the design. MR and CJ analysed the data and MR wrote the final paper in consultation with CJ, JL, and BE.
